# Distance-based topological polynomials and indices of friendship graphs

**DOI:** 10.1186/s40064-016-3271-5

**Published:** 2016-09-15

**Authors:** Wei Gao, Mohammad Reza Farahani, Muhammad Imran, M. R. Rajesh Kanna

**Affiliations:** 1 School of Information Science and Technology, Yunnan Normal University, Kunming, 650500 China; 2 Department of Applied Mathematics, Iran University of Science and Technology, Narmak, Tehran, 16844 Iran; 3 School of Natural Sciences, National University of Sciences and Technology, Sector H-12, Islamabad, P.O. 44000, Pakistan; 4 Department of Mathematical Sciences, United Arab Emirates University, P.O. Box 15551, Al Ain, United Arab Emirates; 5 Post Graduate Department of Mathematics, Maharani’s Science College for Women, Mysore, 570005 India

**Keywords:** Hosoya polynomial, Wiener index, Hyper-Wiener index, Schultz index, Schultz polynomial, Friendship graph, 05C05, 05C12, 05C15, 05C31, 05C69

## Abstract

Drugs and chemical compounds are often modeled as graphs in which the each vertex of the graph expresses an atom of molecule and covalent bounds between atoms are represented by the edges between their corresponding vertices. The topological indicators defined over this molecular graph have been shown to be strongly correlated to various chemical properties of the compounds. In this article, by means of graph structure analysis, we determine several distance based topological indices of friendship graph $F_{3}^{(n)}$ which is widely appeared in various classes of new nanomaterials, drugs and chemical compounds.

## Background

Recent years have witnessed the rapid development in nanomaterials and drugs, which keeps in pace with the development of pharmacopedia. Because of the new issues raised by it, there is a need to test the physical, chemical and biological properties of these new chemical compounds, nanomaterials and drugs, which make the researchers’ workload increased much. Besides, to guarantee the effective results of the research, enough adequate equipment, reagents and human resources are needed to test the performance and the side effects of presented chemical compounds, nanomaterials and drugs. Nevertheless, funds in developing countries (like some countries in Southeast Asia, South America and Africa) are unable to afford the relevant equipment and reagents. Thanks to the contributions from the previous research which discovered that the chemical characteristics of chemical compounds, nanomaterials and drugs and their molecular structures are closely related. Simply speaking, it would benefit the medical and pharmaceutical scientists by providing supports to understand the properties of these chemical compounds, nanomaterials and drugs, if we learn their indicators based on the topological indices. This also helps to make up the experiments shortages. In this way, it can be predicted that the techniques on topological index computation will be welcomed by developing countries by providing the medical and biological information of new chemical compounds, nanomaterials and drugs without chemical experiment conditions.

In the graph computation model, the structure of chemical compounds, nanomaterials and drugs are described as a graph. Every atom are described by an individual vertex, and the chemical bond among them described by the edge. Let *G* be a graph which corresponds to a chemical structure with atom (vertex) set $V(G)$ and chemical bond (edge) set $E(G)$. The distance between vertices *u* and *v,*$d_{G} (u,v)$ or $d(u,v)$, in a graph is the number of edges in a shortest path connecting them and the diameter of a graph *G*, $D(G)$ is the longest topological distance in *G*. The degree $d_{v} (G)$ or *d*_*v*_ of a vertex $v \in V(G)$ is the number of vertices of *G* adjacent to *v*. A vertex $v \in V(G)$ is said to be isolated, pendent, or fully connected if $d_{v} = 0$; $d_{v} = 1$, or $d_{v} = n - 1$, respectively.

A topological index can be described as a real-valued map $f:G \to R^{ + }$ which maps each chemical structure to certain real numbers. For decades, in order to test the features of chemical molecules, some indices like PI index, Wiener index, Harmonic index and Zagreb index are proposed. Meanwhile, some papers also devote to computing these topological indices of special molecular graph in chemical and pharmaceutical engineering.

The Wiener index of *G* was introduced by chemist *Harold Wiener* in 1947 to demonstrate correlations between physicochemical properties of organic compounds and the index of their molecular graphs and is defined as follows (Wiener 1947):

1 $$ W(G) = \frac{1}{2}\sum\limits_{v \in V (G )} {\sum\limits_{u \in V (G )} {d(u,v)} } $$

In 1993 he Hyper-Wiener index is one of distance-based graph invariants, (based structure descriptors), used for predicting physico–chemical properties of organic compounds. The Hyper-Wiener index was introduced by *M. Randić*. The Hyper Wiener index of *G* is defined as follow (Wiener 1948; Randić 1993; Randić et al. 1994):

2 $$ WW(G) = \frac{1}{2}\sum\limits_{v \in V(G)} {\sum\limits_{u \in V(G)} {(d(u,v) + d(u,v)^{2} )} } $$

respectively.

The Hosoya polynomial was first introduced by H. Hosoya, in 1988 (Hosoya 1989) and define as follows:

3 $$ H\left( {G,x} \right) = \frac{1}{2}\sum\limits_{v \in \left( V \right)} {\sum\limits_{u \in \left( V \right)} {x^{{d\left( {u,v} \right)}} } } $$

In references (Polansky and Bonchev 1986; Sridhara et al. 2015; Gao et al. 2016a, b; Gao and Farahani 2016; Schultz 1989; Muller et al. 1990; Gutman and Polansky 1986; Trinajstic 1993; Klavžar and Gutman 1996; Gutman and Klavžar 1997; Hua 2009; Deng 2007; Chen et al. 2008; Zhou 2006), some properties and more historical details of the Wiener and Hyper Wiener indices and the Hosoya polynomial of molecular graphs are studded.

For more details on applications and mathematical properties of this topological based structure descriptor (the Wiener and Hyper-Wiener indices and the Hosoya polynomial) see paper series (Wiener 1948; Randić 1993; Randić et al. 1994; Hosoya 1989; Polansky and Bonchev 1986; Sridhara et al. 2015; Gao et al. 2016a, b; Gao and Farahani 2016).

In 1989, H.P. Schultz introduced a graph theoretical descriptor for characterizing alkanes by an integer number. The “*Schultz molecular topological index*” (MTI) of the graph G is defined as follows (Schultz 1989; Muller et al. 1990)

4 $$ MTI\left( G \right) = \sum\limits_{i = 1}^{N} {[{\mathbf{d}}({\mathbf{A}} + {\mathbf{D}})]_{i} } $$

where two *N* × *N* matrixes **A** and **D** are the adjacency and distance matrixes of *G* (Gutman and Polansky 1986; Trinajstic 1993). ${\mathbf{d}} = (d_{1} ,d_{2} , \cdots ,d_{N} )$ is the $1 \times N$ vector of the degrees of the vertices of *G*. The (*i*, *j*)-th entry of the distance matrix **D**, denoted by *D*_*ij*_, is just the distance between the vertices *i* and *j*, namely the length of a shortest path connecting *i* and *j* (Gutman and Polansky 1986; Trinajstic 1993). Recall that the degree *d*_*i*_ of the vertex *p*_*i*_ is the number of first neighbors of this vertex or, what is the same, the sum of the entries of the *i*-th column of **A**. Note that in the mathematical literature instead of “degree” the name “valency” is sometimes used, which, of course, should be distinguished from valency in chemistry (Klavžar and Gutman 1996; Gutman and Klavžar 1997).

The *Wiener index* (or *Wiener number*) of a connected graph G is equal to the sum of distances between all pairs of vertices of G:

5 $$ W\left( G \right) = \frac{1}{2}\sum\limits_{i = 1}^{N} {\sum\limits_{j = 1}^{N} {D_{ij} } } $$

For the recent results on the Schultz molecular topological index see (Klavžar and Gutman 1996; Gutman and Klavžar 1997; Hua 2009; Deng 2007; Chen et al. 2008; Zhou 2006).The degree distance of G is defined as

6 $$ DD(G) = \frac{1}{2}\sum\limits_{{\left\{ {u,v} \right\} \subset V(G)}} {\left( {d_{u} + d_{v} } \right)d\left( {u,v} \right)} $$

where $d_{u}$ and $d_{v}$ are degrees of vertices *u* and *v* of *G*. The degree distance seems to have been considered first in connection with certain chemical applications by Dobrynin and Kochetova (1994) and at the same time by Gutman (1994) in 1994, who named this degree distance index by the Schultz index. This name was eventually accepted by most other authors [see, e.g., (Zhou 2006; Ilic et al. 2010; Dobrynin 1999; Schultz and Schultz 2000)] and recently we denote the Schultz index of *G* by *Sc(G).*

Later in 1997, *Klavžar* and *Gutman* defined other basic structure descriptors. The modified Schultz index of *G* is defined as:

7 $$ Sc*(G) = \frac{1}{2}\sum\limits_{{\left\{ {u,v} \right\} \subset V(G)}} {\left( {d_{u} \times d_{v} } \right)d\left( {u,v} \right)} $$

Now, there are two topological polynomials of a graph *G* (Gutman 1994) as follows:

8 $$ Sc(G,x)\, = \,\frac{1}{2}\sum\limits_{{\left\{ {u,v} \right\} \subset V(G)}} {\left( {d_{u} + d_{v} } \right)x^{d(u,v)} } $$

and

9 $$ Sc*(G,x)\, = \,\frac{1}{2}\sum\limits_{{\left\{ {u,v} \right\} \subset V(G)}} {\left( {d_{u} \times d_{v} } \right)x^{d(u,v)} } . $$

Obviously,

10 $$ Sc\left( {\text{G}} \right) = \frac{{\partial Sc\left( {\text{G,x}} \right)}}{\partial x}|_{x = 1} $$

and

11 $$ Sc^{*} \left( {\text{G}} \right) = \frac{{\partial Sc^{*} \left( {\text{G,x}} \right)}}{\partial x}|_{x = 1} $$

Several contributions on these indices or related indices can be referred to (Iranmanesh and Alizadeh 2009a, b; Alizadeh et al. 2009; Halakoo et al. 2009; Heydari 2010; Hedyari 2011; Farahani and Vlad 2012; Farahani 2013a, b, c; Farahani 2014; Farahani et al. 2015, 2016; Farahani and Gao 2015; Gao and Farahani 2016; Bokhary et al. 2016; Imran et al. 2016).

The friendship graph is the graph obtained by taking n copies of the cycle graph $C_{3}$ with a vertex in common. It is denoted by $F_{3}^{(n)}$ (Kanna et al. 2016). Friendship graph $F_{3}^{(n)}$ contains $2n + 1$ vertices and $3n$ edges as shown in the figures.

As we mentioned, the new nano materials and drugs are pretty useful in developing areas, and the topological indices are helpful to test the chemical properties of them. In this paper, we present the distance based indices of friendship graph $F_{3}^{(n)}$. The results we obtained here show promising prospects of the application in material and chemical engineering.

## Main results

### **Theorem 1**

*Let*$F_{3}^{(n)}$*be the friendship graph*$\forall n \in {\mathbb{N}} - \left\{ 1 \right\}$*. Then the Hosoya polynomial and the Wiener index of*$F_{3}^{(n)}$*are equal to*:

12 $$ H(F_{3}^{(n)} ,x)\, = \,3nx^{1} + 2n\left( {n - 1} \right)x^{2} $$

*and*

13 $$ W(F_{3}^{(n)} )\, = \,4n^{2} - n. $$

**Fig. 1:**
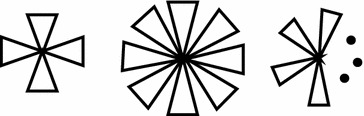
Some examples of friendship graph (in order $F_{3}^{(4)}$, $F_{3}^{(8)}$, $F_{3}^{(n)}$, respectively)

*Proof of Theorem 1* Consider the friendship graph $F_{3}^{(n)}$ deposit in Fig. 1 and be defined as above, with $2n + 1$ vertices and $3n$ edges.

According to Fig. 1 and definition of the friendship graph $F_{3}^{(n)}$ we know that one of center vertex of $F_{3}^{(n)}$ has degree $2n$ and other $2n$ vertices have degree 2. And obviously,

14 $$ \left| {E(F_{3}^{(n)} )} \right|\, = \,\frac{{\left( {1 \times 2n + 2 \times 2n} \right)}}{2}\, = \,n \times \left| {E(C_{n} )} \right|\, = \,3n $$

Also, from Fig. 1 and the edge set of the friendship graph $F_{3}^{(n)}$, one can see that there are $2n$ 1-edge-paths between only center vertex and all other vertices with degree 2 and for all two vertices $v,u \in V(F_{3}^{(n)} )$ with degree 2, there are $n$ 1-edge-paths. Thus the coefficient of the first term of the Hosoya polynomial of friendship graph $F_{3}^{(n)}$ is equal to the number of its edges.

For the second term of the Hosoya polynomial of $F_{3}^{(n)}$, we see that there are $\frac{(2n)(2n - 2)}{2}$ 2-edge-paths between all pair of vertices $v,u \in V(F_{3}^{(n)} )$ with degree 2. So, the coefficient of the second term of the Hosoya polynomial is equal to $2n^{2} - 2n$.

Here, by what have been mentioned above, we have following computations for the Hosoya polynomial of friendship graph $F_{3}^{(n)}$ and alternatively the wiener index of $F_{3}^{(n)}$.

15 $$ H(F_{3}^{(n)} ,x)\, = \,\frac{1}{2}\sum\limits_{{u \in V\left( {F_{3}^{(n)} } \right)}} {\sum\limits_{{v \in V\left( {F_{3}^{(n)} } \right)}} {x^{d(u,v)} } } \, = \,3nx^{1} + 2n\left( {n - 1} \right)x^{2} $$

and

16 $$ W(F_{3}^{(n)} )\, = \,\frac{\partial }{\partial x}H\left( {F_{3}^{(n)} ,x} \right)\,  = \,3n\left( 1 \right) + 2n\left( {n - 1} \right)\left( 2 \right)\, = \,4n^{2} - n. $$

By definition of the Hosoya polynomial of an arbitrary graph *G* with $\left| {V\left( G \right)} \right|$ vertices, it is easy to see that

17 $$ H(G,1) = \left( {_{2}^{{\left| {V\left( G \right)} \right|}} } \right) = \frac{{\left| {V(G)} \right|(\left| {V(G)} \right| - 1)}}{2}. $$

In particular, for $G = \, F_{3}^{(n)}$, it is easy to see that

18 $$ H(F_{3}^{(n)} ,1)\, = 3n+2n(n-1)\, = \,2n^{2} + n\,\left( {_{2}^{2n + 1} } \right) = \frac{(2n + 1)(2n)}{2}\, = \,n(2n + 1) $$

And these complete the proof of Theorem 1.$\square$

### **Theorem 2**

*The Hyper*-*Wiener index of the friendship graph*$F_{3}^{(n)} (\forall n \in {\mathbb{N}} - \left\{ 1 \right\})$*is equals to*:

19 $$ WW(F_{3}^{(n)} ) = 12n^{2} - 6n $$

*Proof of Theorem 2* Consider the friendship graph $F_{3}^{(n)}$ deposit in Fig. 1. By using the above proof and the Wiener index of friendship graph $F_{3}^{(n)}$, we see that

20 $$ \begin{aligned} WW\left( {F_{3}^{(n)} } \right)\, &= \,  \frac{1}{2}\sum\limits_{{{\text{v}} \in {\text{V }}\left( {F_{3}^{(n)} } \right)}} {\sum\limits_{{u \in {\text{V }}\left( {F_{3}^{(n)} } \right)}} {\left( {d\left( {v,u} \right) + d\left( {v,u} \right)^{2} } \right)} } \\ &= \,W\left( {F_{3}^{(n)} } \right){ + }\frac{1}{2}\sum\limits_{{{\text{u,v}} \in {\text{V }}\left( {F_{3}^{(n)} } \right)}} {d\left( {v,u} \right)^{ 2} } \\ &= \,4n^{2} - n + 8n^{2} - 5n \\ &= \,12n^{2} - 6n. \\ \end{aligned} $$

$$\square $$

### **Theorem 3**

*Let*$F_{3}^{(n)}$*be the friendship graph*$(\forall n \ge 2)$. *Then*,

- *The Schultz polynomial of*$F_{3}^{(n)}$*is equal to*
  21 $$ Sc(F_{3}^{(n)} ,x)\, = \,2n(n + 4)x + + 8n(n - 1)x^{2} $$
- *The modified Schultz polynomial of*$F_{3}^{(n)}$*is equal to*
  22 $$ Sc*(F_{3}^{(n)} ,x)\, = \,4n(n + 1)x + + 8n(n - 1)x^{2} $$

*Proof of Theorem 3* Consider the graph of $F_{3}^{(n)}$ depicted in Fig. 1. Using the definition of $F_{3}^{(n)}$ and the results from the proof of Theorem 1, the number of all distinct types of 1 and 2-edge-paths are given in the Table 1. On the other hand, from the definitions of the Schultz and modified Schultz polynomials of a graph $G$, we can obtain the $Sc\left( {G,x} \right)$ and $Sc*\left( {G,x} \right)$ by inserting the coefficient $d_{u + } d_{v}$ and $d_{u} \times d_{v}$ in the Hosoya polynomial.

**Table 1:** The number of all distinct types of 1 and 2-edge-paths

*i*-edge-paths	Degrees of *vertices uv*	Coefficient	Term of Schultz polynomial	Term of modified Schultz polynomial
1	2*n*	2*n*	2*n*(*n*+2)	4*n* ^*2*^
1	22	*n*	4*n*	4*n*
2	2*n*	0	0	0
2	22	2*n*(*n*−1)	8*n*(*n−*1)	8*n*(*n*−1)

Here, we have following computations for the Schultz and modified Schultz polynomials of friendship graph $F_{3}^{(n)}$

23 $$ \begin{aligned} Sc\left( {F_{3}^{(n)} ,x} \right)\, &=  \,\frac{1}{2}\sum\limits_{{u,v \in V(F_{3}^{(n)} )}} {\left( {d_{u} + d_{v} } \right)x^{d(u,v)} } \\ &= \,2n\left( {n + 2} \right)x + 4nx + 0x^{2} + 8n\left( {n - 1} \right)x^{2} \\ &= \,2n\left( {n + 4} \right)x + + 8n\left( {n - 1} \right)x^{2} \\ \end{aligned} $$

24 $$ \begin{aligned} Sc*\left( {F_{3}^{(n)} ,x} \right)\, &= \,  \frac{1}{2}\sum\limits_{{u,v \in V(F_{3}^{(n)} )}} {\left( {d_{u} \times d_{v} } \right)x^{d(u,v)} } \\ &= \,4n^{2} x + 4nx + 0x^{2} + 8n\left( {n - 1} \right)x^{2} \\ &= \,4n\left( {n + 1} \right)x + + 8n\left( {n - 1} \right)x^{2} \\ \end{aligned} $$

Now, the proof of theorem is completed.

### **Theorem 4**

*Let*$F_{3}^{(n)}$*be the friendship graph*$(\forall n \ge 2)$, *then*

- *The Schultz index of the friendship graph*$F_{3}^{(n)} (\forall n \ge 2)$*is equal to*
  25 $$ Sc(F_{3}^{(n)} ) = 2n(9n - 7) $$
- *The modified Schultz index of the friendship graph*$F_{3}^{(n)} (\forall n \ge 2)$*is equal to*
  26 $$ Sc*\left( {F_{3}^{(n)} } \right) = \, 4n\left( {5n - 3} \right). $$

*Proof of Theorem 4* By definitions of the Schultz and modified Schultz indices, we know that

27 $$ \begin{aligned} Sc\left( {F_{3}^{(n)} } \right)\, &= \,\,  \left. {\frac{{\partial Sc(F_{3}^{(n)} ,x)}}{\partial x}} \right|_{x = 1} \\ &= \,\frac{\partial }{\partial x}\left( {2n\left( {n + 4} \right)x + + 8n\left( {n - 1} \right)x^{2} } \right)_{x = 1} \\ &= \,2n\left( {9n - 7} \right). \\ \end{aligned} $$

And also modified Schultz index

28 $$ \begin{aligned} Sc*\left( {F_{3}^{(n)} } \right)\, &= \,  \left. {\frac{{\partial Sc*(F_{3}^{(n)} ,x)}}{\partial x}} \right|_{x = 1} \\ &= \,\frac{\partial }{\partial x}\left( {4n\left( {n + 1} \right)x + + 8n\left( {n - 1} \right)x^{2} } \right)_{x = 1} \\ &= \,4n\left( {5n - 3} \right). \\ \end{aligned} $$

Here, we complete the proof of the Theorem 4.$\square$

## Conclusions

In this article, by means of graph structure analysis, we have determined the several distance-based topological indices of friendship graph $F_{3}^{(n)}$ which is widely appeared in various classes of new nanomaterials, drugs and chemical compounds. These results will be helpful to understand the underlying molecular topologies of these graphs.

## References

[CR1] Alizadeh Y, Iranmanesh A, Mirzaie S (2009). Computing Schultz polynomial, Schultz index of *C*_60_ fullerene by gap program. Digest J Nanomater Bios.

[CR2] Bokhary SA, Imran M, Manzoor S (2016). On molecular topological properties of dendrimers. Can J Chem.

[CR3] Chen S, Jang Q, Hou Y (2008). The Wiener and Schultz index of nanotubes covered by C4. MATCH Commun Math Comput Chem.

[CR4] Deng H (2007). The Schultz molecular topological index of polyhex nanotubes. MATCH Commun Math Comput Chem.

[CR5] Dobrynin AA (1999). Explicit relation between the Wiener index and the Schultz index of cata-condensed benzenoid graphs. Croat Chem Acta.

[CR6] Dobrynin AA, Kochetova AA (1994). Degree distance of a graph: a degree analogue of the Wiener index. J Chem Inform Comput Sci.

[CR7] Farahani MR (2013). Hosoya, Schultz, modified Schultz polynomials and their topological indices of benzene molecules: first members of polycyclic aromatic hydrocarbons (PAHs). Int J Theor Chem.

[CR8] Farahani MR (2013). On the Schultz polynomial, modified Schultz polynomial, Hosoya polynomial and Wiener index of circumcoronene series of benzenoid. J Appl Math Inform.

[CR9] Farahani MR (2013). On the Schultz and modified Schultz polynomials of some harary graphs. Int J Appl Discrete Math.

[CR10] Farahani MR (2014). Schultz indices and Schultz polynomials of harary graph. Pac J Appl Math.

[CR11] Farahani MR, Gao W (2015). The Schultz index and Schultz polynomial of the Jahangir Graphs J5, m. Appl Math.

[CR12] Farahani MR, Vlad MP (2012). On the Schultz, modified Schultz and Hosoya polynomials and derived indices of capra-designed planar benzenoid. Studia UBB Chemia.

[CR13] Farahani MR, Rajesh Kanna MR, Gao W (2015). The Schultz, modified Schultz indices and their polynomials of the Jahangir graphs Jn, m for integer numbers n = 3, m > 3. Asian J Appl Sci.

[CR14] Farahani MR, Rajesh Kanna MR, Gao W (2016). Schultz polynomial of harary graph H_2*r*+1,2*m*+1_. J Chem Biol Phys Sci.

[CR15] Gao W, Farahani MR (2016). Computing the reverse eccentric connectivity index for certain family of nanocones and fullerene structures. J Nanotechnol.

[CR16] Gao W, Farahani MR (2016). Degree-based indices computation for special chemical molecular structures using edge dividing method. Appl Math Nonlinear Sci.

[CR17] Gao W, Wang WF, Farahani MR (2016). Topological indices study of molecular structure in anticancer drugs. J Chem.

[CR18] Gao W, Farahani MR, Shi L (2016). Forgotten topological index of some drug structures. Acta Medica Mediterranea.

[CR19] Gutman I (1994). Selected properties of the Schultz molecular topological index. J Chem Inform Comput Sci.

[CR20] Gutman I, Klavžar S (1997). Bounds for the Schultz molecular topological index of benzenoid systems in terms of the Wiener index. J Chem Inform Comput Sci.

[CR21] Gutman I, Polansky OE (1986). Mathematical concepts in organic chemistry.

[CR22] Halakoo O, Khormali O, Mahmiani A (2009). Bounds for Schultz index of pentachains. Digest J Nanomater Bios.

[CR23] Hedyari A (2011). Wiener and Schultz indices of V-naphtalenic nanotori. Optoelectron Adv Mater Rapid Commun.

[CR24] Heydari A (2010). On the modified Schultz index of *C*_4_*C*_8_(*s*) nanotubes and nanotorus. Digest J Nanomater Bios.

[CR25] Hosoya H (1989). On some counting polynomials in chemistry. Discrete Appl Math.

[CR26] Hua H (2009). Wiener and Schultz molecular topological indices of graphs with specified cut edges. MATCH Commun Math Comput Chem.

[CR27] Ilic A, Klavžar S, Stevanovic D (2010). Calculating the degree distance of partial hamming graphs. MATCH Commun Math Comput Chem.

[CR28] Imran M, Baig AQ, Ali H (2016). On topological properties of dominating David derived networks. Can J Chem.

[CR29] Iranmanesh A, Alizadeh Y (2009). Computing Szeged and Schultz indices of *HAC*_5_*C*_7_*C*_9_[*p*,*q*] nanotube by gap program. Digest J Nanomater Bios.

[CR30] Iranmanesh A, Alizadeh Y (2009). Computing Hyper-Wiener and Schultz indices of *TUZC*_6_[*p*,*q*] nanotube by gap program. Digest J Nanomater Bios.

[CR31] Kanna MR, Kumar RK, Farahani MR (2016). Specific energies of friendship graph. Asian Acad Res J Multidiscip.

[CR32] Klavžar S, Gutman I (1996). A comparison of the Schultz molecular topological index with the Wiener index. J Chem Inform Comput Sci.

[CR33] Muller WR, Szymanski K, Knop JV, Trinajstic N (1990). Molecular topological index. J Chem Inform Comput Sci.

[CR34] Polansky OE, Bonchev D (1986). The Wiener number of graphs. MATCH Commun Math Chem.

[CR35] Randić M (1993). Novel molecular descriptor for structure-property studies. Chem Phys Lett.

[CR36] Randić M, Gou X, Oxley T, Krishnapriyan H, Naylor L (1994). Wiener matrix invariants. J Chem Inform Comput Sci.

[CR37] Schultz HP (1989). Topological organic chemistry 1. Graph theory and topological indices of alkanes. J Chem Inform Comput Sci.

[CR38] Schultz HP, Schultz TP (2000). Topological organic chemistry. 12. Whole-molecule Schultz topo- logical indices of alkanes. J Chem Inform Comput Sci.

[CR39] Sridhara G, Rajesh Kanna MR, Indumathi RS (2015). Computation of topological indices of graphene. J Nanomater.

[CR40] Trinajstic N (1993). Chemical graph theory.

[CR41] Wiener H (1947). Structural determination of paraffin boiling points. J Am Chem Soc.

[CR42] Wiener H (1948). Relations of the physical properties of the isomeric alkanes to molecular structure: surface tension, specific dispersion, and critical solution temperature in aniline. J Phys Chem.

[CR43] Zhou B (2006). Bounds for the Schultz molecular topological index. MATCH Commun Math Comput Chem.

